# Congenital multiple coronary arteriovenous fistulas

**DOI:** 10.1002/ccr3.1716

**Published:** 2018-07-13

**Authors:** Juan Lacalzada‐Almeida, Javier García‐Niebla, María M. Izquierdo‐Gómez, Belén Marí‐López, Ignacio Laynez‐Cerdeña, Francisco Bosa‐Ojeda

**Affiliations:** ^1^ Department of Cardiology Hospital Universitario de Canarias Tenerife Spain; ^2^ Servicios Sanitarios del Área de Salud de El Hierro Valle del Golfo Health Center El Hierro Spain

**Keywords:** coronary arteriovenous fistulas, transthoracic echocardiography

## Abstract

Coronary arteriovenous fistulas are congenital or acquired abnormalities characterized by abnormal communication between the coronary circulation and cardiac chambers or other vessels. Frequently, patients are asymptomatic and their diagnosis can be carried out incidentally by echocardiography. Knowing the echocardiographic findings characteristic of this malformation will prevent the diagnosis from going unnoticed.

## 1 QUESTION

What is the correct patient's diagnosis?

- Patent ductus arteriosus
- Aortopulmonary window
- A Lutembacher syndrome
- Arteriovenous fistula.

Correct answer: D.

**Figure 1:**
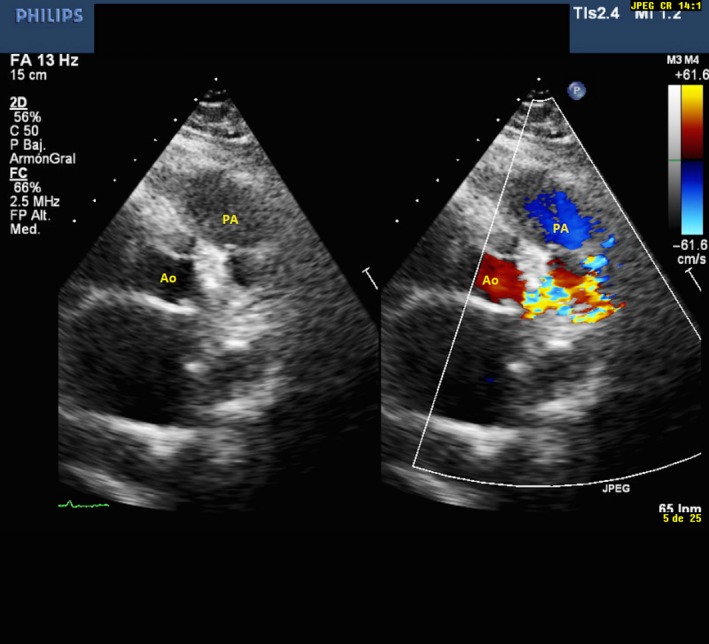
A parasternal short‐axis view of transthoracic echocardiography at the level of the aortic valve (left), showing a turbulent systodiastolic flow, located between pulmonary artery (PA) and aorta (Ao) (right)

**Figure 2:**
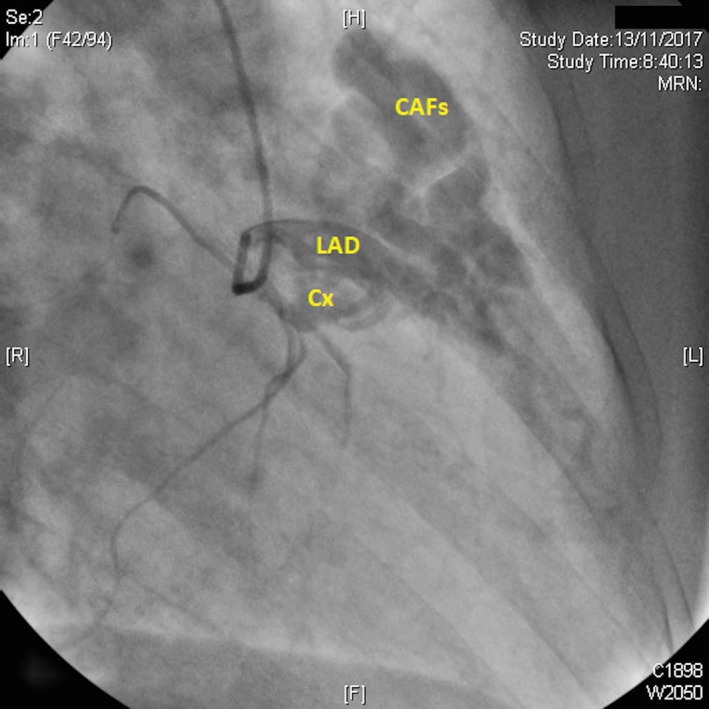
Left coronary angiography showing the presence of two coronary arteriovenous fistulas (CAFs), originating from the left anterior descending (LAD) and circumflex (Cx) coronary arteries draining into the pulmonary artery

**Figure 3:**
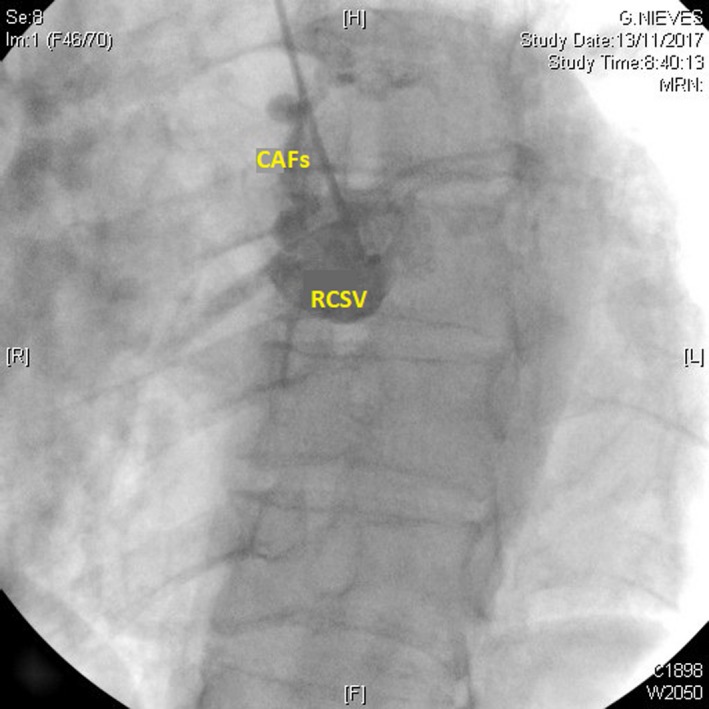
Right coronary angiography showing the presence of the right coronary sinus of Valsalva (RCSV) with a coronary arteriovenous fistula (CAFs) draining into the pulmonary artery

A 62‐year‐old patient was referred to our center for permanent atrial fibrillation to perform a transthoracic Doppler echocardiogram. In the color Doppler, parasternal short‐axis view at the level of the aortic valve, a turbulent systodiastolic flow was found, located between pulmonary artery (PA) and aorta (Ao) (Figure 1 and Video S1). Coronary angiography showed the presence of three coronary arteriovenous fistulas (CAFs), two of them originated from the left anterior descending (LAD) and circumflex (Cx) coronary arteries (Figure 2 and Video S2) and a third from the right coronary sinus of Valsalva (RCSV) (Figure 3 and Video S3), all draining into the PA, with a left‐right shunt with a Qp/Qs of 1.5.

Coronary arteriovenous fistulas have a low incidence in the general population (0.3%‐0.8%), originating from the left and right coronary arteries in 5% of cases and draining into the PA in 17% of cases.^1^ After the diagnosis, an exercise stress echocardiography was performed that was negative for ischemia and showed good functional capacity. Given that the patient was asymptomatic and had no history of heart failure, clinical follow‐up was chosen.

## CONFLICT OF INTEREST

None declared.

## AUTHORSHIP

JLA: involved in conception and design of the work. JGN, MMIG, and BML: wrote the case description and involved in critical revision of the work. ILC: contributed to the description of echocardiography and the key clinical message. FBO: provided the description of the coronary angiography. All authors: involved at each stage of the revision process and contributed substantially to the project's intellectual content.
